# Estimates of HIV-1 within-host recombination rates across the whole genome

**DOI:** 10.1093/ve/veaf052

**Published:** 2025-07-25

**Authors:** Harriet Longley, David Bonsall, Joshua Herbeck, George MacIntyre-Cockett, Sandra E Chaudron, Laura Thomson, Nicholas Grayson, Andrew Mujugira, PANGEA consortium, Christophe Fraser, Jairam Lingappa, Katrina Lythgoe

**Affiliations:** Big Data Institute, Li Ka Shing Centre for Health Information and Discovery, Nuffield Department of Medicine, University of Oxford, Old Road Campus, Oxford, OX3 7BN, United Kingdom; Pandemic Sciences Institute, Nuffield Department of Medicine, University of Oxford, Old Road Campus, Oxford, OX3 7DQ, United Kingdom; Big Data Institute, Li Ka Shing Centre for Health Information and Discovery, Nuffield Department of Medicine, University of Oxford, Old Road Campus, Oxford, OX3 7BN, United Kingdom; Pandemic Sciences Institute, Nuffield Department of Medicine, University of Oxford, Old Road Campus, Oxford, OX3 7DQ, United Kingdom; Wellcome Centre for Human Genetics, Nuffield Department of Medicine, University of Oxford, Old Road Campus, Oxford, OX3 7BN, United Kingdom; Institute for Disease Modelling, Bill & Melinda Gates Foundation, Seattle, WA 98109, United States; Big Data Institute, Li Ka Shing Centre for Health Information and Discovery, Nuffield Department of Medicine, University of Oxford, Old Road Campus, Oxford, OX3 7BN, United Kingdom; Pandemic Sciences Institute, Nuffield Department of Medicine, University of Oxford, Old Road Campus, Oxford, OX3 7DQ, United Kingdom; Wellcome Centre for Human Genetics, Nuffield Department of Medicine, University of Oxford, Old Road Campus, Oxford, OX3 7BN, United Kingdom; Big Data Institute, Li Ka Shing Centre for Health Information and Discovery, Nuffield Department of Medicine, University of Oxford, Old Road Campus, Oxford, OX3 7BN, United Kingdom; Pandemic Sciences Institute, Nuffield Department of Medicine, University of Oxford, Old Road Campus, Oxford, OX3 7DQ, United Kingdom; Wellcome Centre for Human Genetics, Nuffield Department of Medicine, University of Oxford, Old Road Campus, Oxford, OX3 7BN, United Kingdom; Big Data Institute, Li Ka Shing Centre for Health Information and Discovery, Nuffield Department of Medicine, University of Oxford, Old Road Campus, Oxford, OX3 7BN, United Kingdom; Pandemic Sciences Institute, Nuffield Department of Medicine, University of Oxford, Old Road Campus, Oxford, OX3 7DQ, United Kingdom; Big Data Institute, Li Ka Shing Centre for Health Information and Discovery, Nuffield Department of Medicine, University of Oxford, Old Road Campus, Oxford, OX3 7BN, United Kingdom; Pandemic Sciences Institute, Nuffield Department of Medicine, University of Oxford, Old Road Campus, Oxford, OX3 7DQ, United Kingdom; Wellcome Centre for Human Genetics, Nuffield Department of Medicine, University of Oxford, Old Road Campus, Oxford, OX3 7BN, United Kingdom; Department of Global Health, University of Washington Seattle, Seattle, WA 98195, United States; Infectious Diseases Institute, Makerere University, Kampala, P.O. Box 22418 Kampala, Uganda; Big Data Institute, Li Ka Shing Centre for Health Information and Discovery, Nuffield Department of Medicine, University of Oxford, Old Road Campus, Oxford, OX3 7BN, United Kingdom; Pandemic Sciences Institute, Nuffield Department of Medicine, University of Oxford, Old Road Campus, Oxford, OX3 7DQ, United Kingdom; Department of Global Health, Medicine and Paediatrics, University of Washington, Seattle, WA 98195, United States; Big Data Institute, Li Ka Shing Centre for Health Information and Discovery, Nuffield Department of Medicine, University of Oxford, Old Road Campus, Oxford, OX3 7BN, United Kingdom; Pandemic Sciences Institute, Nuffield Department of Medicine, University of Oxford, Old Road Campus, Oxford, OX3 7DQ, United Kingdom; Department of Biology, University of Oxford, South Parks Road, Oxford, OX1 3RB, United Kingdom

**Keywords:** recombination, HIV, intrahost

## Abstract

Recombination plays a pivotal role in generating within-host diversity and enabling HIV’s evolutionary success, particularly in evading the host immune response. Despite this, the variability in recombination rates across different settings and the underlying factors that drive these differences remain poorly understood. In this study, we analysed a large dataset encompassing hundreds of untreated, longitudinally sampled infections using both whole-genome long-read and short-read sequencing datasets. By quantifying recombination rates, we uncover substantial variation across subtypes, viral loads, and stages of infection. We also map recombination hot and cold spots across the genome using a sliding window approach, finding that previously reported inter-subtype regions of high or low recombination are replicated at the within-host level. Importantly, our findings reveal the significant influence of selection on recombination, showing that the presence and success of recombinant genomes is strongly interconnected with the fitness landscape. These results offer valuable insights into the contribution of recombination to evolutionary dynamics and demonstrate the enhanced resolution that long-read sequencing offers for studying viral evolution.

## Introduction

HIV diversifies rapidly within individuals, with genetic diversity in a single-virus population reaching upwards of five percent within a few years of infection in the envelope gene, *env* (Shankarappa et al. 1999). The short viral generation time, rapid turnover of virions, and a high mutation rate—estimated to be on the order of ${10}^{-5}$ mutations/site/generation—generate mutations that enable the virus to continually survive against the host’s immune response (Perelson et al. 1996). Nevertheless, the majority of mutations are deleterious (Zanini et al. 2017), and the recombination of viral genomes is an important mechanism by which deleterious mutations are purged and genomic integrity is preserved (Rawson et al. 2018). In addition, recombination brings together onto the same genetic background beneficial mutations that have been linked to drug resistance, disease progression, and immune-escape (Rambaut et al. 2004), and it has been suggested that recombination accelerates the rate of adaptive evolution (Moradigaravand et al. 2014, Sanborn et al. 2015).

Retroviruses, such as HIV, are diploid, meaning each virion contains two genomic RNA molecules. When a cell is co-infected by two distinct virions, the two different genomic RNAs can be co-packaged into the same virion (Zhuang et al. 2002, Chen et al. 2009). If a subsequent cell is infected by one of these virions, the reverse transcriptase may switch between the two co-packaged genomes during DNA synthesis, with an average two to four switches per replication cycles (Zhuang et al. 2002, Onafuwa et al. 2003). This leads to a recombinant DNA genome that is a mosaic of the two original co-infecting genomes.

There exist numerous software applications for detecting recombination from sequence data (Lemey et al. 2009); however, these methods are typically developed for diverse populations or inter-subtype recombination and are therefore not suitably robust for within-host virus populations where diversity is relatively limited. *In-vitro* methods include the use of retroviral reporter systems (Hu et al. 1997, Chen et al. 2005, 2006), as well as approaches that leverage the genetic differences across subtypes to monitor recombination events (Jetzt et al. 2000, Zhuang et al. 2002, Levy et al. 2004); however, controlled laboratory conditions cannot mimic the complexities of the host environment (Schlub et al. 2010) and any conclusions are further limited due to confounding by factors, including RNA homology (Balakrishnan et al. 2001, 2003).

*In-vivo* approaches better reflect the within-host environment, in particular the dynamics of evolution and recombination under immune pressure. Inevitably, observed recombination in natural infections will underestimate the true recombination rate, as recombination between identical genomes or the generation of a fatal combination of mutations on the same genome is undetectable. Instead, *in-vivo* estimates reflect the *effective recombination rate*, which quantifies the amount of recombination that contributes to the diversity and evolution of the virus population.

Methods can be broadly characterized as breakpoint detection or rate estimation. Breakpoint detection identifies the likely genome location at which template switching has occurred and an array of methodologies and software have been developed specifically for application to viral sequencing data (Jaya et al. 2023). By categorizing regions of above or below average numbers of breakpoints, it has been shown that recombination does not occur randomly across the genome both within and between subtypes (Fan et al. 2007, Archer et al. 2008, Smyth et al. 2014, Jia et al. 2016, Tongo et al. 2018, Grant et al. 2020). Whether hot and cold spots for recombination are consistent across large numbers of individuals and subtypes is not known, and it is difficult to remove the possibility of sequencing artefacts, such as primer locations, that leave a false signal in the data.

In contrast, recombination rate estimation takes a larger scale approach, and the aim is to quantify the frequency at which recombination occurs over a given genome region—typically the entire genome—and provide a continuous measure that is often expressed per generation per genome site. Reported estimates range from ${10}^{-5}$ to $2\times{10}^{-4}$ recombination events/site/generation, suggesting recombination may be occurring even more frequently than point mutations (Shriner et al. 2004, Neher and Leitner 2010, Batorsky et al. 2011, Romero and Feder 2024).

Advances in next-generation deep sequencing have revolutionized the study of within-host dynamics, enabling fine-scale mapping of genetic variation across entire genomes. Given longitudinally sampled sequences, it is now possible to accurately measure decay of linkage disequilibrium (LD) over time. Mutations that initially arise on the same genome exhibit strong LD, but this linkage diminishes as recombination events occur. The rate of LD decay is influenced by the genomic distance between two sites; the greater the distance, the more potential recombination breakpoints exist, leading to a faster decay in linkage. Recent methods leverage the relationship between LD decay and recombination to infer recombination rates, providing deeper insights into viral evolution (Neher and Leitner 2010, Romero and Feder 2024).

The success of a recombinant genome is likely influenced by the fitness landscape of HIV, and functional constraints have been reported to lower the contribution of recombination to viral diversity in *env* (Simon-Loriere et al. 2009). Most recently, high viral load (VL) was shown to be positively associated with the rate of recombination (Romero and Feder 2024), with the hypothesis that a higher VL increases the probability of cell co-infection. Despite the clinical significance of identifying factors that promote or inhibit the effective recombination rate, the small number of individual infections included in most studies has made characterizing the correlates of recombination during natural infection problematic.

Here, we analysed long-read, whole-genome, next-generation sequences from hundreds of individual infections sampled from cohorts of HIV serodifferent African heterosexual couples (one partner living with HIV [PLWH] with their regular sexual partner without HIV at enrolment). The relatively frequent sampling of these individuals meant we could study the dynamics of *in-vivo* recombination with an unprecedented level of detail and resolution. We applied the method of Romero and Feder (2024) for recombination analysis *via* time series linkage decay (RATS-LD), which uncovered significant differences in recombination rate by subtype, VL, infection stage, and genomic position. Importantly, patterns across the genome did not depend upon the sequencing platform (PacBio *vs*. Illumina). The findings of the study contribute to our understanding of how recombination shapes the viral population at both within and between host scales and may provide vital insights for preventing or slowing the emergence of drug resistance and developing successful vaccines.

## Results

### Dataset characteristics

The Partners in Prevention HSV/HIV Transmission (2004–2008) study (Celum et al. 2010), Partners PrEP study (2008–2013) (Baeten et al. 2012), and the Couples Observational Study (2008–2010) (Lingappa et al. 2011) recruited serodifferent couples in East and Southern Africa. Across the three studies, a total of 9 042 couples were longitudinally followed for 1–3 years, with collection of clinical, behaviour, and demographic information as well as samples including blood plasma. Across the three cohorts, HIV sequencing was carried out on plasma from 2 648 couples sampled to include both partners in HIV seroconverting pairs (linked by plasma HIV sequence [see below]), and PLWH in non-transmitting serodifferent couples. Hereafter, individuals who were seropositive at the commencement of the study are termed ‘source’ individuals (*N* = 2 648), irrespective of whether they transmitted the virus to their partner, and seroconverters are termed ‘recipients’ (*N* = 278). Recipients were sampled early in their infection, with the first plasma sample taken typically within weeks of estimated seroconversion time, and all recipients were sampled within the first 6 months following estimated seroconversion (Fig. S1). However, the time since infection for source individuals is not known.

Among couples where HIV seroconversion was identified, targeted regions of plasma HIV genome sequence (regions of *gag* and *env* for Partners in Prevention HSV/HIV Transmission Study and COS, while regions of *gag* and *pol* were sequenced in the Partners PrEP study) were compared between source and recipient to determine epidemiological linkage. We identified 1945 individuals with a combined total of 3 333 samples that had been sequenced on the Illumina platform (Bonsall et al. 2020). Of these, 1 436 samples were sequenced with the PacBio pipeline, 491 of which were analysed in Zhao et al. (2023). A total of 306 individuals had at least two viral samples sequenced with the long-read deep-sequencing PacBio pipeline, representing 163 sources and 143 recipients. A secondary dataset was generated to allow for verification of the results of the analysis. This consisted of individuals with at least two viral samples of sufficient depth sequenced with the Illumina pipeline, a minority of whom also featured in the primary dataset. In total, 356 individuals were included in the Illumina dataset, of which 171 individuals also appear in the PacBio dataset.

Log-transformed VL measurements were approximately normally distributed with a mean of $4.79\ {\log}_{10}$ copies per ml. Source individuals exhibited a significantly lower average VL compared to recipients (*t*-test of log-transformed VLs, *P*-value $1.3\times{10}^{-5}$, mean of $4.90\ {\log}_{10}$ copies per ml in recipients *vs*. $4.66\ {\log}_{10}$ copies per ml in sources). Higher VLs in recipients are likely due to recipients being captured earlier into reflection, with some recipients captured towards the end of acute infection. Infections were predominantly classified as Subtype A1 (53%), with 18% identified as Subtype D, 18% as Subtype C, and the remaining 11% largely composed of circulating recombinant forms (CRFs) and unique recombinant forms (URFs).

The recombination rate, defined as the number of recombination events per site per viral generation, was inferred from linkage decay over time between alleles at pairs of diverse sites using the PacBio sequencing data. We applied the RATS-LD method which exploits the relationship between LD and recombination, by measuring the rate at which linkage decays between two sampling points as a function of the product of time and genomic distance. Source individuals contributed the majority (~95%) of site pairs due to the higher genetic diversity observed later in infection, supporting the interpretation that source individuals were typically at a more advanced stage of infection than recipients. Overall, the dataset encompasses 775 467 unique site pairs, with 33% located in *env*, 27% in *pol*, and 13% in *gag*, and 28% in other regions of the genome. Approximately 25% of the pairs of sites in the secondary Illumina dataset (216 007 unique pairs) also appeared in the primary PacBio dataset.

### Measured recombination saturates across long genomic distances and timescales

The length of sequencing reads significantly impacts the resolution and accuracy of detecting linkage decay between alleles, with longer reads capturing genetic variants that are positioned further apart on the same genome, providing a more comprehensive view of how linkage decays over distance. The time between sampling points is also important, with the higher the number of generations the lower the resolution at which recombination can be measured. This is due to saturation caused by multiple recombination events between pairs of sites between time points. Here, we used the relatively long reads produced from PacBio sequencing, and we also benefited from the relatively frequent sampling of individuals, and plasma was sampled from study participants approximately every 3 months.

Although our aim is to quantify the *effective recombination rate*, which captures the recombination that contributes to viral population diversity, the constraints imposed by sequence length and time between sampling make this challenging. Instead, we report the *measured recombination rate*, which is an outcome of recombination, evolutionary processes (e.g. selection), and linkage decay. We first tested the expectation that both read length and time between sampling affect measured rates of recombination by varying the time-scaled distance threshold. Time-scaled distance, $d\Delta t,$ is the product of the number of generations over which we measure linkage and the genomic distance between the sites, and the threshold is the maximum possible value of this product. A lower threshold therefore excludes pairs of sites that are either genomically close but have sampling points spread far apart in calendar time or sites that are distant on the genome but have sampling points closely spaced in time.

**Figure 1:**
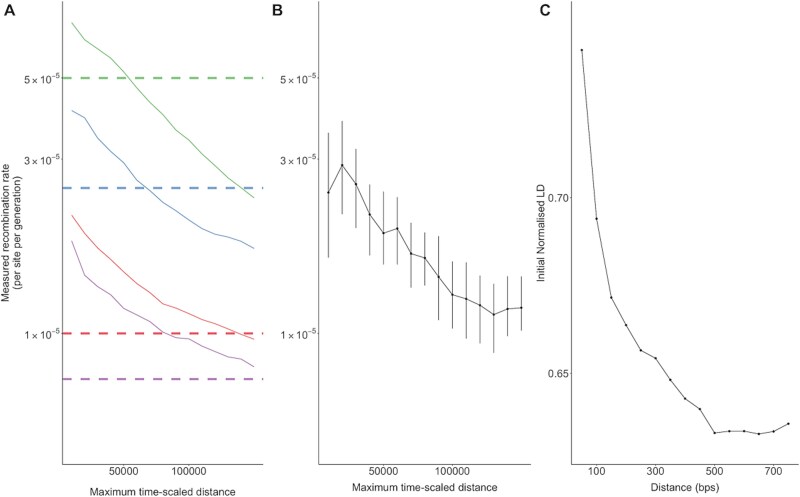
Recombination rate decreases with time-scaled distance. (A) The recombination rate/site/generation calculated by the RATS-LD method (Romero and Feder 2024) with sequences representing 30 simulated virus populations evolving over time (Haller and Messer 2023). Four recombination rates (/site/generation) values were considered (〖5 × 10〗^(−5)^ in green, 〖2.5 × 10〗^(−5)^ in blue, 10^(−5)^ in red, and 〖7.5 × 10〗^(−6)^ in purple, and are shown in this order going top to bottom on the figure). The dashed lines indicate the true recombination rate fixed as a parameter in the simulation. As the maximum time-scaled distance increases, the inferred recombination rate reduces. The best choice of cut-off for time-scaled distance for accurate estimation depends upon the magnitude of the true rate of recombination. (B) The whole-genome recombination rate for increasing maximum time-scaled distance threshold decreases for the PacBio sequences dataset. Time-scaled distance is the product of the genomic distance between two sites and number of generations between sampling points. Error bars indicate 95% confidence intervals generated from 100 bootstrapped replicates. (C) The initial normalized LD (LD standardized by the maximum possible value) of a pair of sites given the genomic distance between sites for the PacBio sequence dataset. Data are binned into groups spanning 50 bps, ranging from 0–50 bps to 700–750 bps, and the mean linkage of the group is denoted by a point. The decrease in LD plateaus at 500 bps, and linkage at sites situated >500 bps likely reflects residual background linkage and are not informative for recombination estimation.

First, we analysed 30 simulated populations with known recombination rates using the package SLiM (Haller and Messer 2023). As the maximum value of $d\Delta t$ increased, the measured recombination rate decreased (Fig. 1A). When increasing the choice of the true rate of recombination, shorter distances between sites and/or shorter time intervals between sampling are required to accurately recover the true recombination rate. Repeating the analysis on the PacBio sequencing dataset, we observed a similar relationship between the estimated genome-wide recombination rate and the maximum value of $d\Delta t$ (Fig. 1V).

This relationship likely occurs because as the time-scaled distance increases, recombination reaches a point of saturation where the linkage between alleles at two sites is completely broken down. Beyond this point, further increases in distance or number of generations do not reveal additional recombination events because the alleles are already effectively unlinked. This saturation can artificially lower the measured recombination rate as the decay of LD plateaus. The distance at which sites can be in significant linkage along the HIV genome has previously been determined to be lower than 200 bps (Neher and Leitner 2010, Zanini et al. 2015); however, we found linkage continued to decay as distance increased until plateauing at around 500 bps, at which point the linkage estimates reflect residual background LD that is not dependent upon distance and is therefore not informative for recombination estimation (Fig. 1C). To account for the maximum number of base pairs over which we observe linkage decaying with distance, and a mean number of ~150 generations between time points, we apply a maximum value of $d\Delta t=75\ 000$ for our remaining analyses, with the exception of analysis on short-read Illumina data which has a maximum of $d\Delta t=50\ 000$. Although measured recombination rates are sensitive to the data factors, and the optimal maximum time-scaled distance is uncertain when the true recombination rate is unknown, consistent distance thresholds, or appropriate adjustments can still provide valuable insights into the factors influencing recombination.

### Recombination rate varies by viral load, subtype, and stage of infection

**Figure 2:**
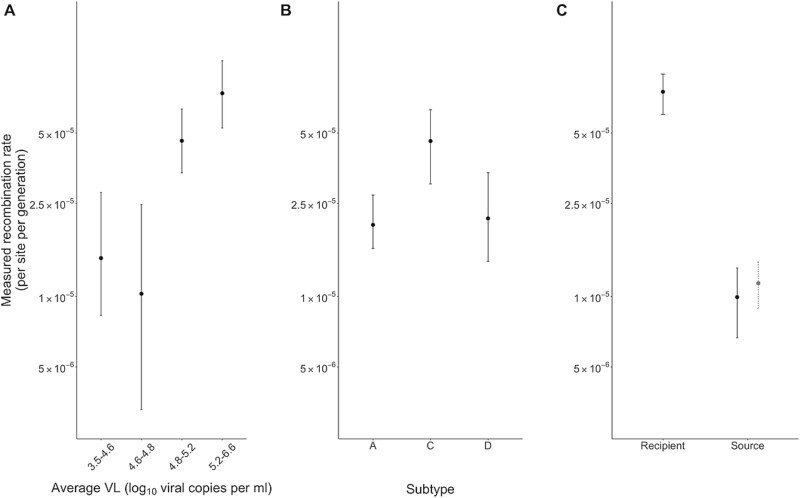
Average recombination rate varies by viral load, subtype, and stage of infection. (A) The recombination rate/site/generation by viral load group for all infections with viral load measures taken on the same day as plasma sampling. The median of bootstrapped replicates is represented by a point and 95% confidence intervals are represented by bars. Every pair of sites has a viral load value that is the average of the viral load across the two time points that contribute to the pair. The entire set of viral load measures are ordered by ascending value and split into quantiles, each containing the same number of observations. In agreement with Romero and Feder (2024), recombination estimates increase with viral load, with an elevated recombination rate observed in infections with an average viral load greater than 10^4.8^. (B) The rate of recombination by subtype after adjusting for differences in viral load. The median and confidence intervals of the bootstrap replicates are represented as they are in (A). Subtype C has a higher rate than subtypes a or D. (C) The recombination rate for the recipient *versus* the source group. The median and confidence intervals of the bootstrap replicates are represented as they are in (A). For individuals sampled later into infection (i.e. sources), the rate is significantly lower than in individuals sampled within the first 12–18 months following infection (i.e. recipients). To account for higher viral loads and more frequent sampling in recipients, the source dataset was subsampled such that the viral load and number of days between samples matched that of recipients. The point with the dashed confidence interval denotes the unadjusted recombination rate/per site/per generation.

It has been previously observed in a small cohort of 10 individuals that the recombination rate varied by VL both within and between individuals, with high VL (>$4.9\ {\log}_{10}$ copies per ml) associated with elevated rates (Romero and Feder 2024). It was hypothesized that elevated rates at higher VLs were driven by an increase in the frequency of cell co-infection. We performed a similar analysis on our dataset to verify the finding in a large independent dataset. VL measures were grouped into quantiles of increasing value, with each group containing an equal number of pairs of sites. We found substantial variation across the groups, with high VL (>$4.8\ {\log}_{10}$ copies per ml) associated with a larger genome-wide recombination rate estimate (Fig. 2A), corresponding closely to the existing estimate of the VL values at which recombination rates were elevated (Romero and Feder 2024). Median recombination rates increased with VL: 3.5–4.6 ${\log}_{10}$ copies per ml: $1.46\times{10}^{-5}\ \left(\mathrm{CI}:0.83\times{10}^{-5}-2.78\times{10}^{-5}\right)$/site/generation, 4.6–4.8 ${\log}_{10}$ copies per ml: $1.03\times{10}^{-5}\ \left(\mathrm{CI}:0.33\times{10}^{-5}-2.47\times{10}^{-5}\right)$/site/generation, 4.8–5.2 ${\log}_{10}$ copies per ml: $4.63\times{10}^{-5}\ (\mathrm{CI}:3.37\times{10}^{-5} -6.22\times{10}^{-5})$/site/generation, 5.2–6.6 ${\log}_{10}$ copies per ml: $7.39\times{10}^{-5}\ \left(\mathrm{CI}:5.25\times{10}^{-5}-10.2\times{10}^{-5}\right)$/site/generation. Non-overlapping confidence intervals between high and low VL groups indicate that these differences are unlikely due to sampling variability alone.

The measured rate of recombination also varied by subtype (Fig. 2B). The highest genome-wide recombination rate was observed in the Subtype C dataset ($4.62\times{10}^{-5}\ (\mathrm{CI}:3.03\times{10}^{-5}-6.29\times{10}^{-5})$/site/generation), while Subtypes A and D had approximately equal rates of $2.02\times{10}^{-5}\ \left(\mathrm{CI}:1.61\times{10}^{-5}-2.71\times{10}^{-5}\right)$/site/generation and ($2.16\times{10}^{-5}\ \left(\mathrm{CI}:1.14\times{10}^{-5}-0.34\times{10}^{-5}\right)$/site/generation), respectively. To test whether variation in VL explained the elevated rates in Subtype C infections, we compared the distributions of log-transformed VLs across the three datasets, however, no significant differences were found (*t*-test, *P* > .05 for each comparison). There are several biological and epidemiological factors that could explain the differences in the rates of recombination, including differences in replication dynamics, cell tropism differences that affect the likelihood of multiple infection of a cell, or viral genetic differences that affect template switching frequency. Further research is needed to disentangle the possible mechanisms behind subtype-specific measured recombination rate differences.

To investigate whether the effective recombination rate is a dynamic quantity that varies over the course of infection, we compared rate estimates across source and recipient groups. The evolutionary dynamics between the groups are likely to be distinctive due to the level of adaptation towards the host immune response—recipients were sampled within the first 12 to 18 months of infection, while source individuals had been infected for several years. The measured recombination rate in recipients was $7.5\times{10}^{-5}\left(\mathrm{CI}:6.0\times{10}^{-5}-8.9\times{10}^{-5}\right)$/site/generation, while the measured recombination rate in the source group was substantially lower at $1.1\times{10}^{-5}\left(\mathrm{CI}:8.9\times{10}^{-6}-1.4\times{10}^{-5}\right)$/site/generation (Fig. 2C). This reduction in the effective recombination rate may be attributed to multiple factors. Firstly, the differences in selection pressure acting for and against recombinants may drive this pattern. In early infection, the developing immune response may create conditions where recombinants can establish at higher frequencies before being purged, while in chronic infection, a more adapted immune system may rapidly eliminate novel recombinants. Similarly, greater diversity in later infection may lead to a greater proportion of recombination events producing deleterious combinations of mutations that are removed from the population, and we therefore do not observe these recombination events. Alternatively, higher rates of recombination may be driven by differences in target cell availability—the larger pool of activated CD4+ T cells during early infection may facilitate higher rates of cellular co-infection.

We repeated the analysis using the Illumina dataset (Fig. S2). Increasing recombination rates with VL, elevated recombination in recipients and the ordering of subtype rates were all supported.

### Substantial variation in rates of recombination between and within genes

When the recombination rate is inferred from pairs of sites across the entire genome (i.e. genome wide), the median number of events/site/generation is ~$1.51\times{10}^{-5}$ (CI: $1.39\times{10}^{-5}-2.01\times{10}^{-5}$), which falls within the range of several other *in-vivo* estimates (Shriner et al. 2004, Neher and Leitner 2010, Batorsky et al. 2011, Romero and Feder 2024). However, when the genome is divided into specific genes and protein domains, significant fluctuations in recombination rates are observed (Table 1). The recombination rates for the entire *gag* and *pol* genes are relatively similar, at $1.26\times{10}^{-5}$ and $1.30\times{10}^{-5}$ recombination events/site/generation, respectively, however, *pol* contains protein domains with exceptionally high recombination rates, with the rate in the p31 integrase domain exceeding the average by more than three-fold.

**Table 1:** The recombination rate /site/generation by gene or protein domain. The difference to the genome average is calculated as a ratio

Gene	HXB2 coordinates	Recombination rate ($\times{10}^{-5}$)	Ratio difference to genome median
gag	790–2 292	1.26 (CI: 0.95–1.61)	0.83
P17	790–1 186	1.66 (CI: 0.55–4.03)	1.1
P24	1 186–1879	1.51 (CI: 0.91–2.36)	1
pol	2085–5 096	1.30 (CI: 0.97–1.65)	0.86
prot	2 253–2 550	1.21 (CI: 0.85–1.79)	0.8
p51 RT	2 550–3 870	1.10 (CI: 0.66–1.65)	0.73
p15 RNase	3 870–4 230	3.64 (CI: 1.79–7.19)	2.41
p31 int	4 230–5 096	5.64 (CI: 1.83–12.45)	3.74
vif	5 041–5 619	0.51 (CI: 0.23–1.20)	0.34
vpr	5 559–5 850	3.15 (CI: 1.90–4.93)	2.09
vpu	6 062–6 310	6.81 (CI: 1.61–16.62)	4.52
env	6 225–8 795	1.63 (CI: 0.96–3.24)	1.08
gp120^*^	6 225–7 758	2.25 (CI: 0.91–6.06)	1.49
gp41	7 758–8 795	1.45 (CI: 0.68–3.35)	0.96

^*^Variable loops are excluded from the analysis and therefore only 70% of the protein nucleotides are included.

**Figure 3:**
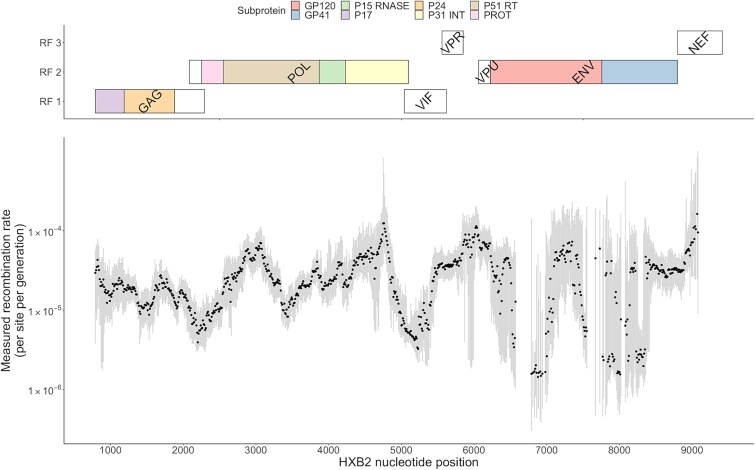
Recombination rate/site/generation for overlapping sliding windows of length 500 bps, moving in increments of 10 bps. Genes and reading frames are described above. Shaded grey bars represent a 95% confidence interval calculated by bootstrap replicates. Proteins and subproteins are denoted above. Windows in which the lower end of the CI was negative were discarded, of which the large majority were located in env. Within env, confidence intervals are often notably wide, likely due to challenges in sequence alignment and a high frequency of insertions and deletions relative to the HXB2 reference, which may impact time-scaled distance estimates. The patterns of hot/cold spots were replicated across subtypes and sequencing platform (Fig. S3) and 10 previously identified hot/cold spots identified at an inter-subtype level (Jia et al. 2016) are also found to be hot/cold spots in our analysis in 9 of the 10 cases (Table S1).

To better understand the drivers of inter- and intra-gene variation, we applied a sliding window approach to quantify the relative recombination rate across the genome (Fig. 3). The range of rate estimates was very wide, spanning as low as $1.43\times{10}^{-6}$ (HXB2: 6630–7130) up to $1.24\times{10}^{-4}$ (HXB2: 4510–5010) recombination events/site/generation a difference of 100-fold. Consistent with previous observations (Fan et al. 2007, Jia et al. 2016, Grant et al. 2020), we found that *env* is flanked by two short regions of frequent recombination -*tat/vpu* and the C1 region of the 5′ end of the gene and *rev/tat* exons at the 3′ end of the gene—while the measured rate of recombination varies substantially within the gene. A short region between the second and third variable loops is notable for a particularly low frequency of recombination, where the recombination rate observed in the genome windows with midpoints spanning HXB2: 6 790–6 980 falls below $1.6\times{10}^{-6}$. Regions of high and low frequency of recombination were consistent across subtype and were also evident in the Illumina dataset (Fig. S3).

The method encountered limitations in certain regions of *env*, particularly those in close proximity to the variable loops, where some windows yielded either wide confidence intervals or negative median rates across bootstraps. These technical challenges likely stem from two factors: the substantial difficulty in accurately aligning the highly diverse *env* sequences across time points and infections, and the high frequency of insertions and deletions relative to the HXB2 reference sequence, which may distort time-scaled distance measurements by artificially inflating genetic distances.

The degree of sequence homology can also limit recombination, with regions of higher diversity expected to feature a lower number of breakpoints *in-vivo*. To test whether sequence diversity drives variation across the genome, we tested window-specific recombination events/site/generation with window-specific diversity measures. Diversity is defined as the average hamming distance of the sequences of a specific sample from the consensus sequence of the sample, with the average hamming distance of a window taken as the average (median) diversity score across all individuals. There was no evidence of a decrease in the number of recombination events/site/gen as diversity increased, and rather a significant positive correlation was observed, albeit of a very small magnitude (Fig. S4).

Analyses of breakpoints in URFs and CRFs have produced descriptions of the distribution of hot and cold spots across the genome at the inter-subtype level; however, it remains unclear whether inter-subtype breakpoints align with the recombination hot and cold spots observed within a single-virus infection. Using data from Jia et al., we compared windows with high and low numbers of breakpoints identified in CRFs and URFs circulating in Africa to the patterns observed in our analysis (Jia et al. 2016), and all five inter-subtype hot spots identified by Jia et al. exhibited above-average recombination rates in our analysis, and four of the five cold spots had below average rates (Table S1).

### Recombinants selected against in *env*

**Figure 4:**
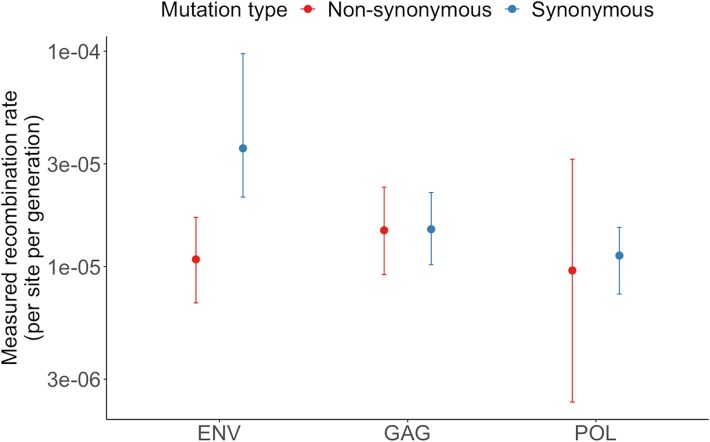
Substantial differences in recombination rate in *env* by mutation type. The recombination rate is estimated separately by mutation type (non-synonymous *vs*. synonymous) of *env*, *gag* and *pol*. Only pairs of sites at which the change is frequency between time point is low (<10%) are included to account for the effects of strong directional selection on non-synonymous sites on linkage estimates. Pairs of sites included in the ‘non-synonymous’ group are restricted to pairs where the selection of the minor allele at both sites would result in a protein-level change, assuming no nucleotide substitutions at other sites within the same codon. Error bars represent 95% confidence intervals generated by bootstrap replicates. In *env*, we propose that a lower rate of measured recombination in pairs of sites at which there is a non-synonymous change signifies selection against recombinants.

Selection and recombination are intrinsically linked: recombination events that create deleterious combinations of mutations are likely to be purged by selection, effectively reducing the measured recombination rate. To disentangle these processes, we estimated gene-level recombination rates (*gag*, *pol*, *env*) based on whether the minor allele would result in a synonymous or non-synonymous substitution, assuming the other two positions in the codon remained unchanged. To minimize the influence of strong directional selection on non-synonymous mutations, we first focused on pairs of sites where allele frequency changes across time points were low, defined as an absolute frequency change of <10% (Fig. 4). In *gag* and *pol*, recombination rates did not differ substantially between synonymous and non-synonymous sites (*gag* non-synonymous: $1.45\times{10}^{-5}\left(\mathrm{CI}:0.97\times{10}^{-5}-2.46\times{10}^{-5}\right)$/site/generation, synonymous: $1.55\times{10}^{-5}\left(\mathrm{CI}:1.00\times{10}^{-5}-2.20\times{10}^{-5}\right)$/site/generat-ion; *pol* non-synonymous: $1.06\times{10}^{-5}\left(\mathrm{CI}:0.23\times{10}^{-5}-2.89\times{10}^{-5}\right)$, synonymous: $1.11\times{10}^{-5}\left(\mathrm{CI}:0.77\times{10}^{-5}-1.53\times{10}^{-5}\right)$). In contrast, *env* showed a significant difference between the two groups: the recombination rate between synonymous sites was over three times higher than between non-synonymous sites (*env* non-synonymous: $1.15\times{10}^{-5}\left(\mathrm{CI}:0.67\times{10}^{-5}-1.9\times{10}^{-5}\right)$, synonymous: $3.57\times{10}^{-5}\left(\mathrm{CI}:2.08\times{10}^{-5}-11.3\times{10}^{-5}\right)$) with significance demonstrated by non-overlapping 95% confidence intervals. We also repeated the analysis using all sites regardless of frequency change (Fig. S5) and found the same qualitative pattern—higher recombination between synonymous sites in *env*. This observation suggests that functional constraints in *env* generate epistatic interactions between non-synonymous mutations, reducing the fitness of recombinants carrying certain combinations.

## Discussion

Recombination is a key mechanism driving the remarkable adaptability of HIV, facilitating the emergence of beneficial mutation combinations while purging deleterious ones (Lemey et al. 2009). In this study, we applied a time-series linkage decay method to a large cohort of individual infections to determine the detailed patterns of recombination across the genome. It has been previously demonstrated that hot and cold spots for recombination exist: consistent hot and cold spots within the *pol* and *gag* regions have been reported across multiple infections (Smyth et al. 2014), and recombination hot spots have been repeatedly described in studies of inter-subtype recombination (Magiorkinis et al. 2003, Fan et al. 2007, Jia et al. 2016, Grant et al. 2020) and in experimental settings (Zhuang et al. 2002, Simon-Loriere et al. 2010). Recombination patterns are therefore likely to be intrinsic to the genome structure and governed by underlying mechanisms that operate broadly across individuals. We provide strong evidence that recombination breakpoint locations are indeed systematic, with significant correlations in window-specific rates observed across subtypes and sequencing platforms, implying consistency in regions of high and low recombination. We also confirmed that regions identified as recombination hot or cold spots at the inter-subtype level (Jia et al. 2016) also exhibit similar patterns within individual hosts infected with a single subtype. This finding suggests that the formation of URFs and CRFs is at least partially driven by within-host recombination dynamics. Consequently, it may be possible to predict future emerging recombinant forms based on within-host recombination patterns.

By considering variation across the entire genome with a sliding window approach, we have identified novel hot and cold spots, including a strong hot spot within the p31 region, which encodes the integrase protein in the *pol* gene. The protein is of significant clinical importance, as it is the target of integrase strand transfer inhibitors (Smith et al. 2021). Our study cohort are treatment naïve; however, this finding suggests a natural susceptibility to recombination in this region, which could increase the likelihood of drug resistance mutations emerging over time.

The measurable frequency of recombination events in natural infection partly reflects evolutionary processes—primarily selection pressure—and rate variation within and between genes is very likely influenced by the fitness of recombinant genomes. In agreement with previous studies, we observed a low rate of measurable recombination in regions of *gp120*, a protein that is required for cell entry and recombination is likely to be suppressed due to functional constraints (Simon-Loriere et al. 2009). Additionally, interdependencies between *gp120* and *gp41* proteins have been said to lower the fitness of recombinant genomes (Golden et al. 2014, Woo et al. 2014, Bagaya et al. 2015). By partitioning sequence data by mutation type, we showed linkage was much more rapidly broken down between pairs of sites at which a substitution with the minor allele is synonymous in comparison to pairs where a substitution is non-synonymous. Infrequent recombination in *env* is therefore likely the result of strong epistasis and selection against recombinants, emphasizing the disruptive potential of recombination in virus adaptation and a probable large discrepancy in the true frequency of recombination with measurable recombination *in-vivo,* as well as the role of interacting and interdependent mutations in the evolutionary success of mutations in *env.* More broadly, we hypothesize that the reduction in the measurable recombination rate in individuals sampled later in infection reflects the interaction between recombination and selection. We propose that the virus, having become well-adapted to the host environment after lengthy infection, rapidly purges deleterious recombinants, hence lowering the measured rate of recombination. Additionally, the increased genetic diversity characteristic of long-term infection likely generates a landscape where recombination more frequently disrupts co-adapted genetic elements, resulting in fewer recombinants reaching detectable frequencies. These temporal dynamics highlight recombination’s dual role as both a diversification mechanism and a target of selection during HIV’s evolutionary trajectory within hosts.

Our study is unique due to the large sample size, the use of long-read deep sequencing from the PacBio platform, and the replication of results with a dataset built on sequences generated on the Illumina platform. With LD measures from sequences outputted from long-read PacBio and short-read Illumina platforms, we have shown that key findings are not a consequence of the sequencing process or noise, and that they are replicated in a dataset of pairs of sites with minimal overlap and a largely separate set of infections. By leveraging the read length of PacBio sequences, we considered the effect that distance and time has on recombination estimation, as previous proposals for the range at which linkage is observed (100–200 bps) have been limited by read length and relatively sparse sampling (Neher and Leitner 2010, Zanini et al. 2015). By applying the method to simulated data, we found that the required read length for accurate rate estimation depends on sampling frequency and the true recombination rate, with more frequent sampling necessitating longer reads to accurately capture linked mutations before recombination occurs. In the empirical dataset, LD continued to decay with increasing genetic distance up to 500 bps, indicating that LD can be seen to persist over longer distances if sampling is sufficiently frequent. Consequently, careful consideration of the necessary read length based on the sampling regime is crucial for analysing linkage or conducting phylogenetic analyses, and comparisons of LD patterns across different datasets should be made cautiously. Indeed, in certain regions of the genome with a low number of recombination breakpoints, the analysis may be overestimating the rate of recombination. Nonetheless, when controlling for time-scaled distance, LD decay methods can still provide valuable insights into the viral and host factors that influence the effective recombination rate.

In summary, our findings reveal substantial variation in recombination rates across the genome that are consistently replicated across subtypes, sequencing platforms, and at the inter-subtype level, suggesting an intrinsic viral property driving recombination dynamics. The contribution of recombination to within-host populations is shown to be strongly tied to selection processes, emphasizing the need for *in-vivo* studies and highlighting the role of strong epistasis in *env.* Ultimately, understanding the recombinogenic properties of HIV and recombination dynamics can guide the development of treatments and vaccines that avoid viral escape strategies.

## Methods

### Study cohort

All individuals whose samples were analysed were enrolled in one of three African HIV serodifferent heterosexual couples studies, the Partners in Prevention HSV/HIV Transmission study (Lingappa et al. 2009, Celum et al. 2010), Partners PrEP study (Mujugira et al. 2011, Baeten et al. 2012), and the Observational Cohort Study (Lingappa et al. 2011), all of which recruited serodifferant couples in subSaharan Africa and followed them for between 12 and 36 months. ‘Source’ individuals were living with HIV-1 when recruited, and ‘recipient’ individuals were initially HIV-1 negative and seroconverted at some point during the study. The estimated date of seroconversion of a recipient is taken as the midpoint between the last negative HIV test and the first positive HIV test. Participants were followed up quarterly, with the exception of recipients in the Partners PrEP study and sources in the Partners in Prevention HSV/HIV Transmission study who were followed up with study drug monthly. In accordance with the recruitment criteria, participants were not on antiviral treatment when enrolling and did not begin any treatment during the course of the study. All participants gave written informed consent for the storage of samples and future HIV genetic studies. The study was registered with ClinicalTrials.gov and approved by the University of Washington Human Subjects Review Committee, as well as the ethics review committees at any institutions collaborating with individual study sites.

### Sample preparation, sequencing, and processing

Samples were processed using the veSEQ-HIV protocol (Bonsall et al. 2020) which has been validated for drug resistance testing on the Illumina platform (Jenkins et al. 2023). Briefly, total RNA was extracted from plasma, and DNA sequencing libraries were prepared with the SMARTer Stranded Total RNA-Seq—Pico-Input Mammalian Kit v2. Unfragmented RNA was reverse transcribed using adapter-linked random hexamers, followed by conversion into double-stranded dual-indexed DNA libraries with 12 polymerase chain reaction (PCR) cycles. Libraries were pooled and size-fractionate with AMPure-XP beads, adjusting the polyethylene glycol (PEG) ratios to produce a low molecular weight and high molecular weight fraction for sequencing. For Illumina sequencing the cutoffs for low-molecular weight (LMW) and high-molecular weight (HMW) material was 0.5 and 0.68 PEG:pool (*v:v*), and for PacBio cut offs were 0.68 and 0.8 PEG:sample pool (*v:v*)*.* The HMW and LMW fractions were re-pooled at a molar ratio of 1:9 for subsequent HIV enrichment. Custom HIV-specific biotinylated probes captured HIV DNA fragments using the IDT hybridization wash kit. Following capture, libraries were amplified for sequencing with 12 PCR cycles, ensuring sufficient yield while minimizing amplification bias. Where stated, captured pooled libraries were sequenced on Illumina NovaSeq using the P2 500 cycle flow cell. For PacBio sequencing, captured material was ligated to SMRT-bell PacBio adapters and loaded onto a Sequel II SMRT Cell (8 M) for sequencing on a PacBio Sequel IIe instrument.

Illumina reads were processed with Kraken to remove human and bacterial reads (Wood and Salzberg 2014), followed by trimming with Trimmomatic (Bolger et al. 2014). Contigs were assembled using SPAdes (Bankevich et al. 2012) and metaSPAdes (Nurk et al. 2017) with default parameters, and clustered with cd-hit-est (Fu et al. 2012). Reads were mapped to sample-specific references, and consensus sequences were generated using Shiver (Wymant et al. 2018a)*.* For samples without assembled contigs, reads were mapped to the closest matching genome from the Los Alamos HIV database (http://www.hiv.lanl.gov). The same pipeline was adapted for PacBio HiFi reads, which were demultiplexed using PacBio LIMA, and a dummy reverse complement-read was synthesized to mimic paired-end data for shiver.

All individuals included in the study had at least two longitudinal samples that have been sequenced and subsequently processed with Shiver. The primary analysis included only PacBio long reads, while a secondary dataset, consisting of Illumina short reads, was assembled to further verify the findings.

The software Phyloscanner (Wymant et al. 2018b) was used to generate sliding windows of alignments for each individual. Windows covered the whole genome from position 520 (with respect to HXB2 sequence). The reads in every window were aligned to HXB2 positions, and so insertions and deletions relative to HXB2 were excluded from the analysis. For the primary dataset of long-read sequences, a window length of 750 bps was chosen to balance the need for both long reads to measure the effect of distance on linkage measures and the need for sufficient depth to accurately quantify allele frequencies. For the secondary dataset, a window length of 250 bps was chosen as the Illumina platform produces short reads. A window needed a depth >10 reads to be included in either dataset. For any genomic site included in the analysis at time *t,* the minor variant must have a frequency of 5% or greater at time *t* to be included.

For each sample included in the dataset, the subtype, country, sample timing with respect to the earliest time point, and sequencing platform are detailed in Supplementary Data File 1. In total, 1 207 samples were analysed in this study. In the main study dataset (PacBio sequencing platform), 783 samples were included. In the sensitivity analysis dataset (Illumina sequencing platform), 865 samples were included. A total of 441 samples were included that were sequenced by both Illumina and PacBio. Additional metadata can be supplied upon application to the PANGEA consortium (see Data Availability).

### Subtype classification

The HIV-1 subtype was determined by the subtype of the best reference selected by Shiver during the sequence processing stage. For the subtype-specific analyses, only Subtypes A1, C, and D were included as other subtypes were insufficiently represented. Samples where the best reference subtype was not consistent across samples were not included in the subtype-specific datasets. As a result, 43 individuals were removed from the main analysis of subtype effects, and 45 individuals were removed from the subtype analysis of the Illumina dataset. In total for the PacBio dataset, 141 individuals were included in the Subtype A analysis, 33 individuals in the Subtype C dataset, and 48 in the Subtype D analysis. The corresponding figures for the Illumina dataset were 159, 40, and 71.

### Viral loads

The VL associated with a ${D}_{\mathrm{ratio}}^{\prime }$ is the mean of the individual’s VL at time $t$ and time $t+\Delta t$. If a VL measure was not available for the same date that the virus sampling took place, the data point was removed from any analysis where filtering by VL was necessary. As a result, in the PacBio dataset, 221 847 pairs of sites had a reported VL viral, and in the Illumina dataset 105 838 pairs had a reported VL value.

### Linkage disequilibrium calculation

For each window of sequences outputted by Phyloscanner, allele frequencies at every position were calculated. Any position at which the proportion of gaps was greater than 10% was excluded, and to be included in the recombination rate analysis the minor allele frequency (MAF) must exceed 5%. One individual was removed from the analysis as the number of diverse sites was exceptionally high, suggestive of possible super infection, sequencing error, or contamination. For all such sites, LD was calculated for each pair of sites *(X, Y*) in the window. The standardized LD metric is defined as the ${D}^{\prime }$ statistic:


$$ {D}^{\prime }=\frac{\left|{p}_{\mathrm{A}\mathrm{B}}-{p}_{\mathrm{A}}{p}_{\mathrm{B}}\right|}{D_{\mathrm{max}}} $$

where allele A and allele B are the major alleles at position *X* and *Y*, respectively, ${p}_{\mathrm{A}}$ is the frequency of allele A, ${p}_{\mathrm{B}}$ is the frequency if allele B, ${p}_{\mathrm{AB}}$ is the frequency of the haplotype AB, and ${D}_{\mathrm{max}}$ is the maximal possible value of linkage given ${p}_{\mathrm{A}}$ and ${p}_{\mathrm{B}}$. For a pair of sites (*X*,*Y*) sampled at time *t* to be included in the recombination analysis, all four possible haplotypes must be observed. Additionally, the standardized LD metric for a pair of sites (*X*,*Y*) at the earliest included time point must exceed 0.2. LD calculations were performed in Python 3.7.

### Recombination analysis

RATS-LD is a method developed by Romero and Feder (2024) that exploits the relationship between linkage, recombination, and distance and time. In brief, mutations that appear in the population on the same background are initially in high LD, and the rate at which linkage decays as a result of recombination is related to the physical distance between the sites at which the mutations appeared. The RATS-LD method assumes that between time *t* and time $t+\Delta t$, the linkage between allele A and allele B decays by the equation:


$$ {D}^{\prime}\left(t+\Delta t\right)={D}^{\prime }(t){e}^{-\rho d\Delta t} $$

where *d* is the distance between sites, $\Delta t$ is the number of generations between time points and $\rho$ is the recombination rate/site/generation. The RATS-LD method calculates the metric, ${D}_{\mathrm{ratio}}^{\prime },$ for each pair of sites across two time points, defined as:


$$ {D}_{\mathrm{ratio}}^{\prime }=-\log \left(\frac{D^{\prime}\left(t+\Delta t\right)}{D^{\prime }(t)}\right)=\rho d\Delta t $$

This equation implies a linear relationship between ${D}_{\mathrm{ratio}}^{\prime }$ and $d\Delta t$ with $\rho$. However, to account for the effect of small differences arising from sampling errors, as well as the effect of other evolutionary forces for large value of $d\Delta t$, the method expresses the relationship between linkage decay and time-scaled distance in the functional form:


$$ f(x)={c}_0+{c}_1\left(1-{e}^{-{c}_2x}\right) $$

Near zero, the slope approaches $\rho$, and so the estimate of the recombination rate/site/generation, $\hat{\rho}$, is given by


$$ \hat{\rho}={f}^{\prime}\left(x=0\right)={c}_1{c}_2 $$

The calculation of ${D}_{\mathrm{ratios}}^{\prime }$ and fitting of the curve $f(x)$ were performed in R v4.2.1. For a full explanation of RATS-LD and validation of the method with simulations considering recombination rates, selection effects, and population size, refer to Romero and Feder (2024).

As the pairs of sites are identified from overlapping windows of 750 bp reads over the genome, the same pair of sites can appear in the dataset multiple times. To account for this, the mean ${D}_{\mathrm{ratio}}^{\prime }$ for a given pair of sites and timepoints is included in the dataset for curve fitting. Sites within the variable loops V1–V5 were excluded from the analysis as the method performed poorly in these regions, most likely due to alignment issues.

Confidence intervals were generated by bootstrapping. We found that the intervals typically stabilized beyond 100 replicates and increasing further was computationally prohibitive. Bootstrapping was performed at an individual level, such that for an individual with *n* pairs of sites, *n* pairs would be sampled with replacement for curve fitting. Recombination analysis was performed in R version 4.2.

### Matching by viral load and sampling time

To account for the effect of differences in VL when comparing recombination rates by stage of infection (recipient *vs*. source), we adjusted datasets such that the VL distributions approximately matched. We matched VL distributions between comparison groups through stratified subsampling. First, we divided the reference distribution (i.e. recipient infection) into quintiles, each containing 20% of observations. We then subsampled from the comparison group (i.e. source infection) within each quintile boundary to achieve proportionally matched distributions across the entire VL range. As source individuals were sampled less frequently, the subsampled source dataset was subsampled again to match the distribution of sampling timing in the recipient dataset. The same approach as was applied to VL was applied to the distance subsampling.

### Recombination estimates by sliding windows

To identify local and global patterns in recombination rates, a sliding window approach was taken. For analyses of the main dataset, the recombination rate of windows of 500 bps length with overlaps of 450 bps was estimated. The width was set as 500 bps in order to balance the need for distantly spaced sites with a sufficient number of data points to fit in the model. A window was excluded if the 95% CI included 0, i.e. the CI covered negative values. Sliding window rates were also measured for subtype-level comparison and for the Illumina dataset, where a window length of 750 bps length was chosen to account for the reduction in the total number of pairs in the analysis.

### Estimation of correlation for window-specific rates

We calculated the Pearson correlation coefficient for the median recombination rate at each 750 bp genome window, with three comparisons in total: Subtype A *versus* Subtype C, Subtype A *versus* Subtype D, and Illumina *versus* PacBio. By considering the correlations between windows, we are measuring the extent of similarity in patterns of hot and cold spots for recombination along the genome across subtypes. To test the statistical significance of the correlation, we performed permutation tests. The window IDs for one subtype were shuffled and a correlation test was performed. We repeated the permutated correlation calculation 10 000 times to provide a distribution of coefficients to compare against the correlation coefficient of the non-permutated dataset in order to test for statistical significance of the observed correlation. To determine the windows that differed significantly across subtypes, we performed a linear regression of Subtype A window rates at window *i* against Subtype C/D window rates at position *i.* Outliers were defined as windows at which the standardized residual was >2.

### Mutation type classification

For each genome site in each infection, we determined whether the minor allele at the site can be classified as non-synonymous (i.e. leads to a change at the amino acid level) or synonymous (i.e. no change at the amino acid level). When determining whether a nucleotide substitution would result in an amino acid substitution for each site, we assumed that the two other nucleotides in the codon did not change. We used the standard genetic code for HIV-1 translation, with each site being analysed in the context of its reading frame in the appropriate gene. In the case of overlapping reading frames, the sequences were translated for each reading frame and a mutation was classified as non-synonymous if in any of the reading frames there was an amino acid substitution. For sites with multiple minor alleles, we classified the site based on the most frequent minor allele. Insertions and deletions were excluded from this classification analysis.

### Diversity metric

For each infection, we analysed sequence diversity within sliding windows using the latest sampled time point. Within each window, we calculated the Hamming distance (proportion of nucleotide mismatches) between every read and the consensus sequence of that window. The diversity measure for each window in a given infection was defined as the mean Hamming distance across all reads. To obtain a representative diversity measure for each genomic window across the entire dataset, we calculated the median of these per-infection diversity values.

### Simulations

As was presented in Romero and Feder (2024), simulations of neutrally evolving populations were generated in SLiM (Haller and Messer 2023), with a mutation rate of ${10}^{-5}$ and an effective population size of ${10}^4$. Generated sequences were 1000 bps long and simulations ran for $5\times{10}^4$ generations, after which 100 sequences were sampled every 50 generations for 600 generations. The recombination rate for each simulation was fixed, ranging from $7.5\times{10}^{-6}$ to $5\times{10}^{-5}$ recombination events/site/generation. For each choice of recombination rate, 30 simulations were performed, and ${D}_{\mathrm{ratios}}^{\prime }$ for the outputted sequences were pooled.

## Supplementary Material

New-supp-figure_1-virus_evolution_SM_veaf052

suppTable1_veaf052

SM_1_veaf052

SM_2_veaf052

## Data Availability

Consensus sequences of Illumina samples are available at https://github.com/PANGEA-HIV/PANGEA-Sequences, with sample IDs in the Supplementary Data File 2 (SM2). Further data including metadata (participant age, sex, VL) and deep-sequencing data can be requested by contacting the PANGEA consortium www.pangea-hiv.org. The data and scripts for the simulation analyses and scripts for the analysis pipeline of the PANGEA sequencing data are available at https://github.com/HLongleyOx/RecombinationHIV
