# Supplementary material for: Estimates of HIV-1 within-host recombination rates across the whole genome
Source: Virus Evol. 2025 Jul 25;11(1):veaf052. doi: 10.1093/ve/veaf052 (PMC12309388; doi:10.1093/ve/veaf052)
Supplement: New-supp-figure_1-virus_evolution_SM_veaf052 [file new-supp-figure_1-virus_evolution_sm_veaf052.docx]

Supplementary Material


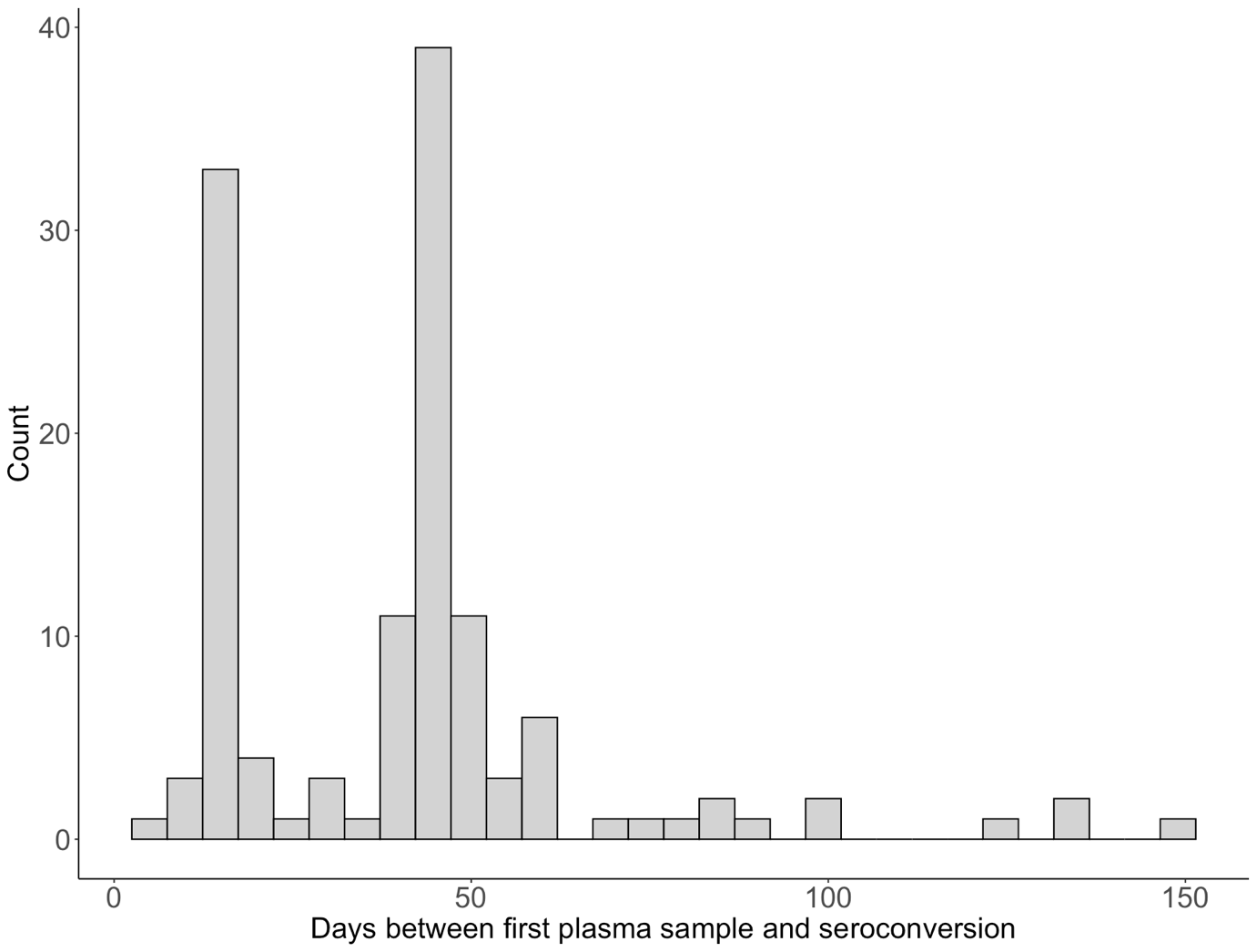


**Figure 1: Estimated time between seroconversion and first plasma sample time for recipients.** The estimated seroconversion time is taken as the midpoint between the last negative HIV test and the first HIV positive test. For all recipients, the first plasma sample time is within 5 months of estimated seroconversion time.


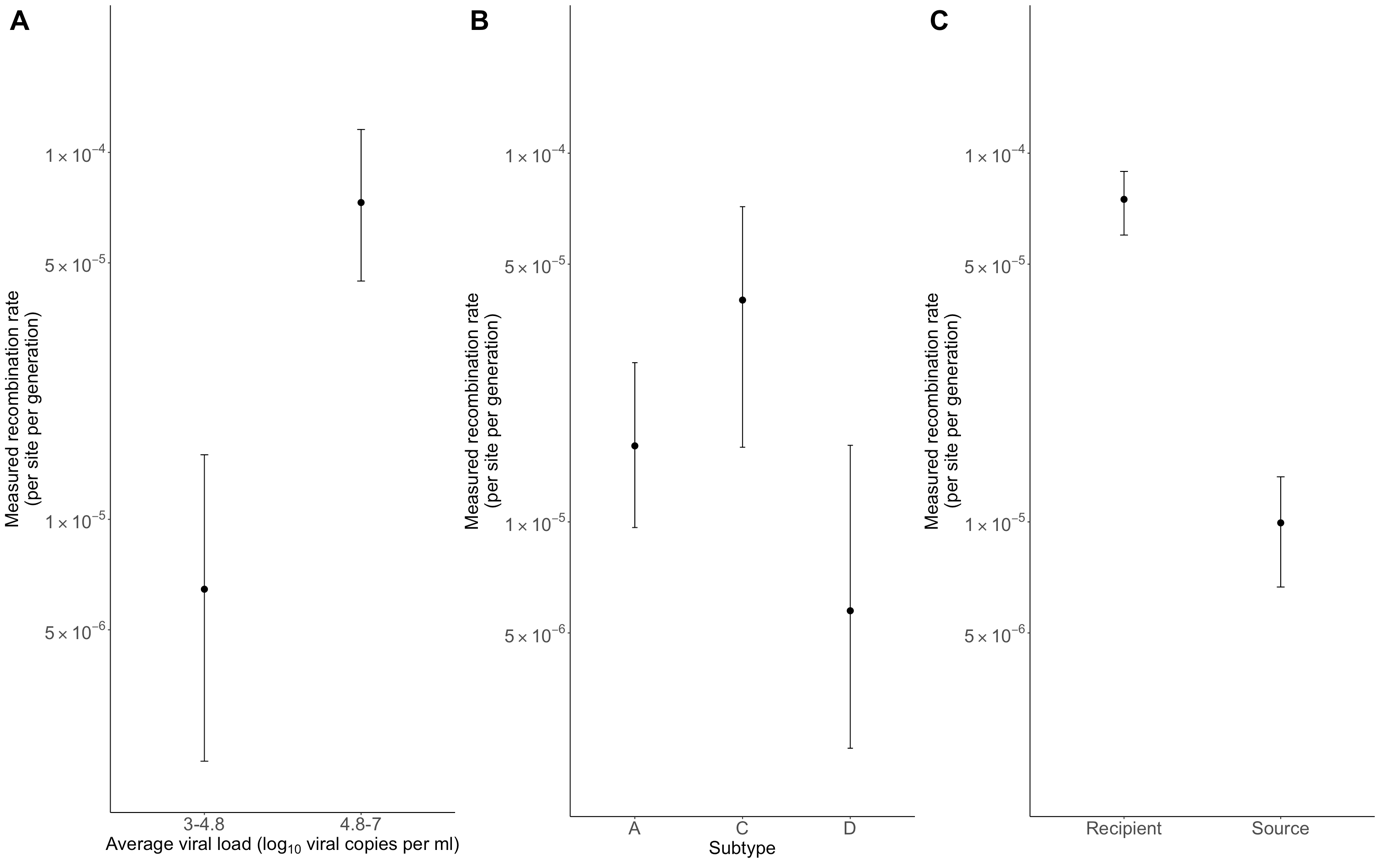


**Figure 2: The whole-genome recombination rate varies by viral load, subtype and stage of infection for the Illumina dataset. A)** The whole genome recombination rate increases with viral load. Viral load measures were split into three groups of equal size representing low, medium and high recombination rates. Five groups were used in the main analysis, however due to the smaller size of the Illumina dataset three groups were chosen. Higher viral load measures are associated with an increase in the whole-genome recombination rate. **B)** The whole-genome recombination rate by subtype. As in the main analysis, subtype C is found to have the highest rate, however the differences are not statistically significant due to overlapping CIs. **C)** Whole genome recombination rate by stage of infection. As was observed in the PacBio analysis, the recombination rate is substantially lower in individuals sampled later into infection compared to individuals sampled in the first 12-18 months. As in the main analysis, datasets were subsampled to match on viral load and sampling frequency.


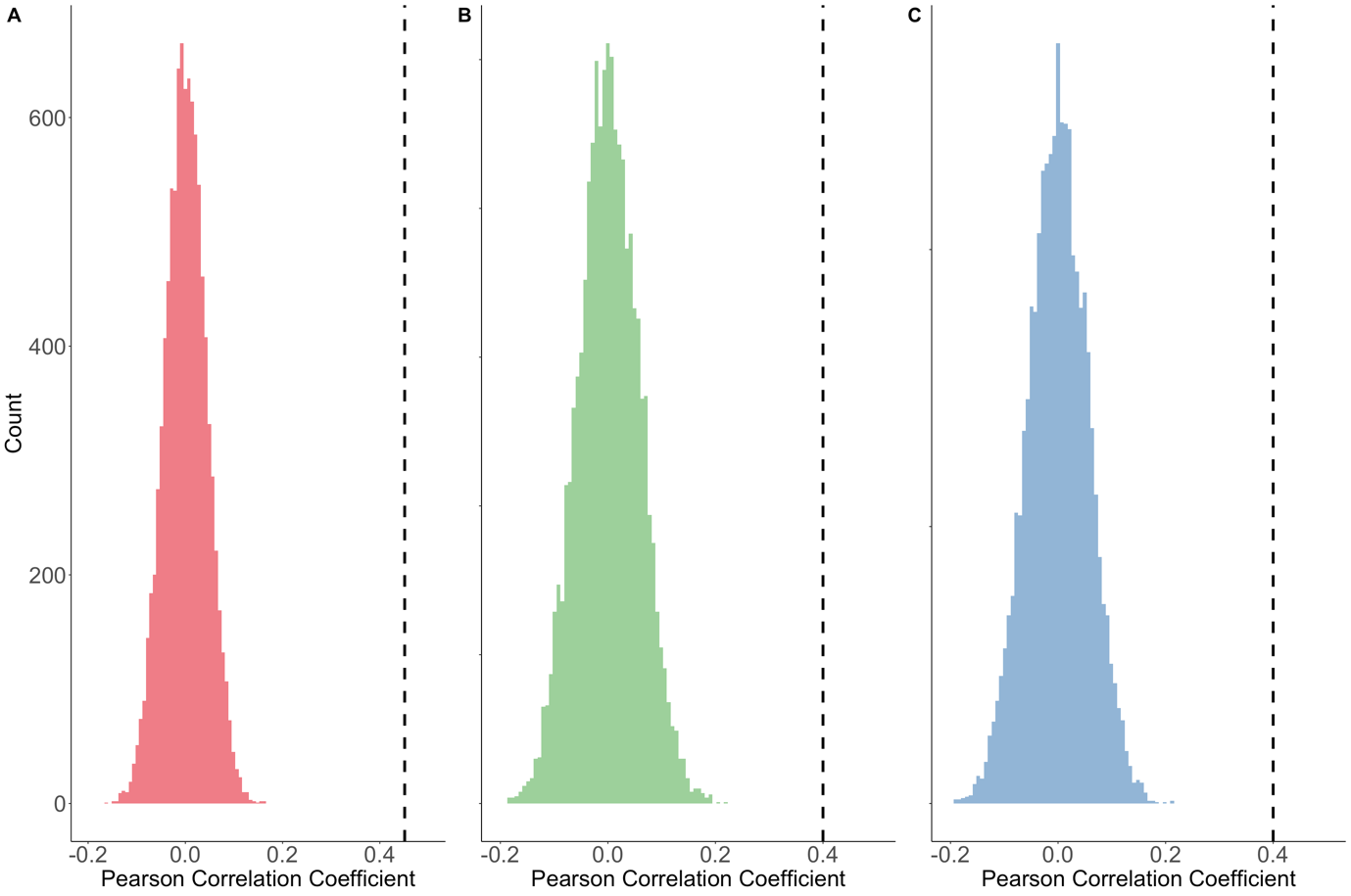


**Figure 3: Significant correlation coefficients between sliding windows for Illumina and subtype-specific datasets with the entire PacBio dataset. A)** Correlation test result for comparison between PacBio and Illumina datasets. The histogram represents correlations of 10,000 replicates of a permutated dataset where the window coordinates of the illumina sliding window rates are shuffled, and dotted lines represent the correlation calculated from the unshuffled dataset which is significantly higher than the permuted dataset, indicating significant correlation in genome patterns of recombination across the two subtypes. **B)** Correlation tests results for subtype A vs subtype C sliding window output. **C)** Correlation test results for subtype A vs subtype D.


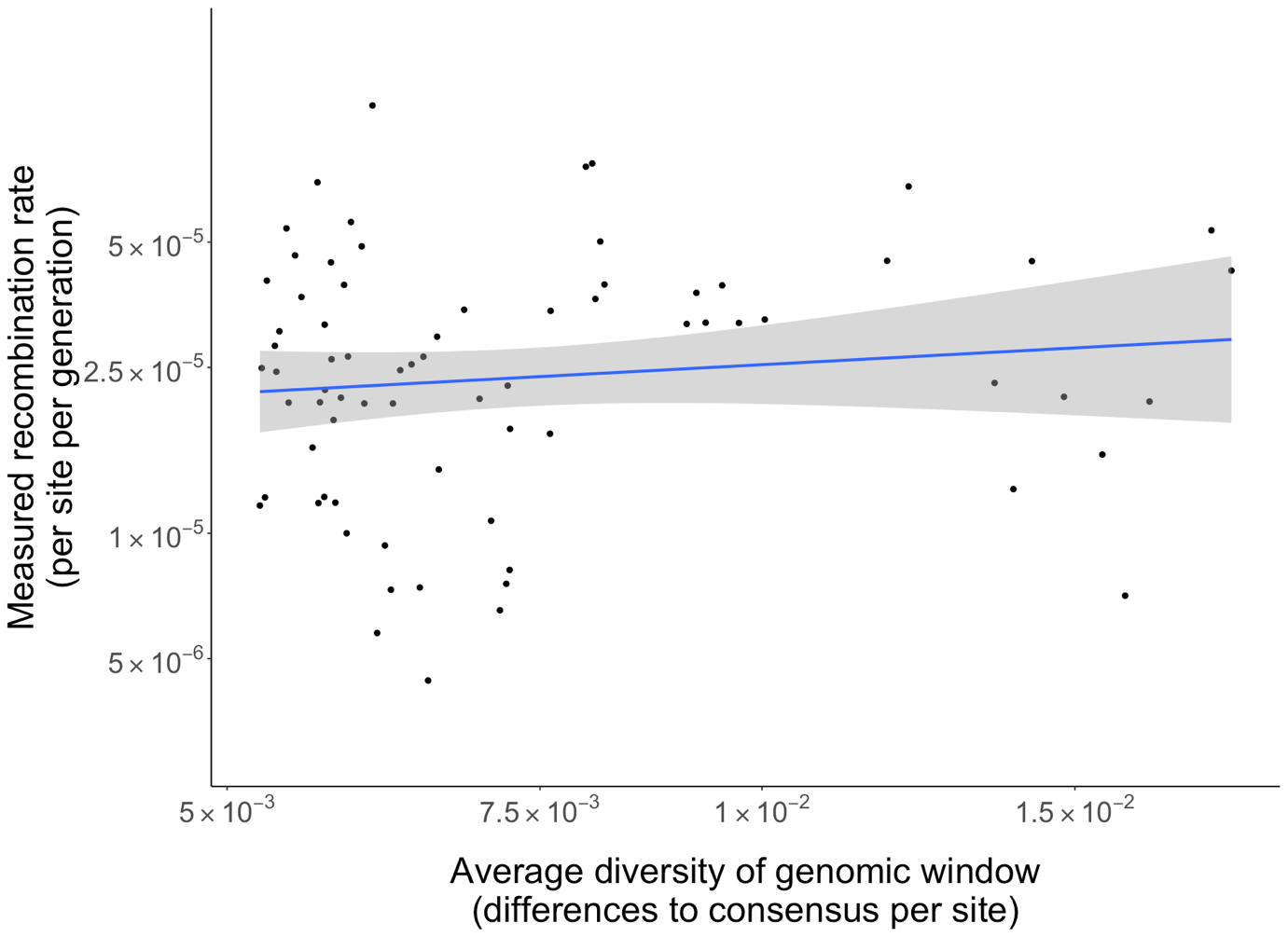


**Figure 4: Relationship between window-specific diversity and window-specific recombination rate.** Recombination rates per site per generation were inferred using the RATS-LD method for each sliding window. Diversity was quantified as the average Hamming distance to the consensus sequence across all individuals, using only the latest sample per infection. Both diversity and recombination rate values were log-transformed. Linear regression and Pearson correlation tests revealed a statistically significant (p < 0.05) but very weak positive association, indicating no evidence for a decrease in recombination rate with increasing diversity.

**Table 1:** The recombination rate per site per generation for hot and cold spots identified in unique recombinant forms and circulating recombinant forms seen in Africa (Jia et al. 2016). To account for distance effects on rate estimation, we calculated comparison values for each region by restricting the genome-wide measure to pairs of sites within distances equivalent to the width of each region of interest. For the window spanning positions 790-990, the model did not yield a positive rate, likely due to poor model fit resulting from minimal evidence of recombination in this region, as was also the case for the lower bound of the CI for 7790-8190. To assess statistical significance, we compared hotspot rates to the upper bound of the confidence interval for the genome-wide median rate, and coldspot rates to the lower bound of the corresponding confidence interval. All regions showed significantly different values from genome-wide estimates (higher for hotspots and lower for coldspots) with the exception of the region spanning positions 3890-3990.

| HXB2 start | HXB2 end | Region type | Median rate (95% CI) **(** $\boldsymbol{\times10}^{\boldsymbol{-5}}\boldsymbol{)}$ | Genome-wide median rate (95% CI) **(** $\boldsymbol{\times10}^{\boldsymbol{-5}}\boldsymbol{)}$ | Lower/higher than genome average |
| --- | --- | --- | --- | --- | --- |
| 790 | 990 | Cold spot | -1.1 (-28--0.38) | 2.1 (1.6-2.7) | TRUE |
| 1290 | 1690 | Cold spot | 0.54 (0.27-0.81) | 1.8 (1.3-2.4) | TRUE |
| 3890 | 3990 | Cold spot | 11 (3.6-22) | 3.3 (2.3-4.9) | FALSE |
| 5140 | 5340 | Cold spot | 1 (0.48-1.7) | 2.1 (1.6-2.7) | TRUE |
| 7790 | 8190 | Cold spot | 0.48 (-106-3) | 1.8 (1.3-2.4) | TRUE |
| 1090 | 1189 | Hot spot | 12 (3.5-20) | 3.3 (2.2-5.6) | TRUE |
| 3190 | 3289 | Hot spot | 8.7 (3.9-16) | 3.3 (2.2-5.6) | TRUE |
| 4090 | 4289 | Hot spot | 6 (2.7-10) | 1.9 (1.4-2.5) | TRUE |
| 5890 | 6389 | Hot spot | 6.9 (4.1-9.6) | 2 (1.5-2.4) | TRUE |
| 8290 | 9090 | Hot spot | 3.5 (2.3-5) | 1.9 (1.5-2.3) | TRUE |


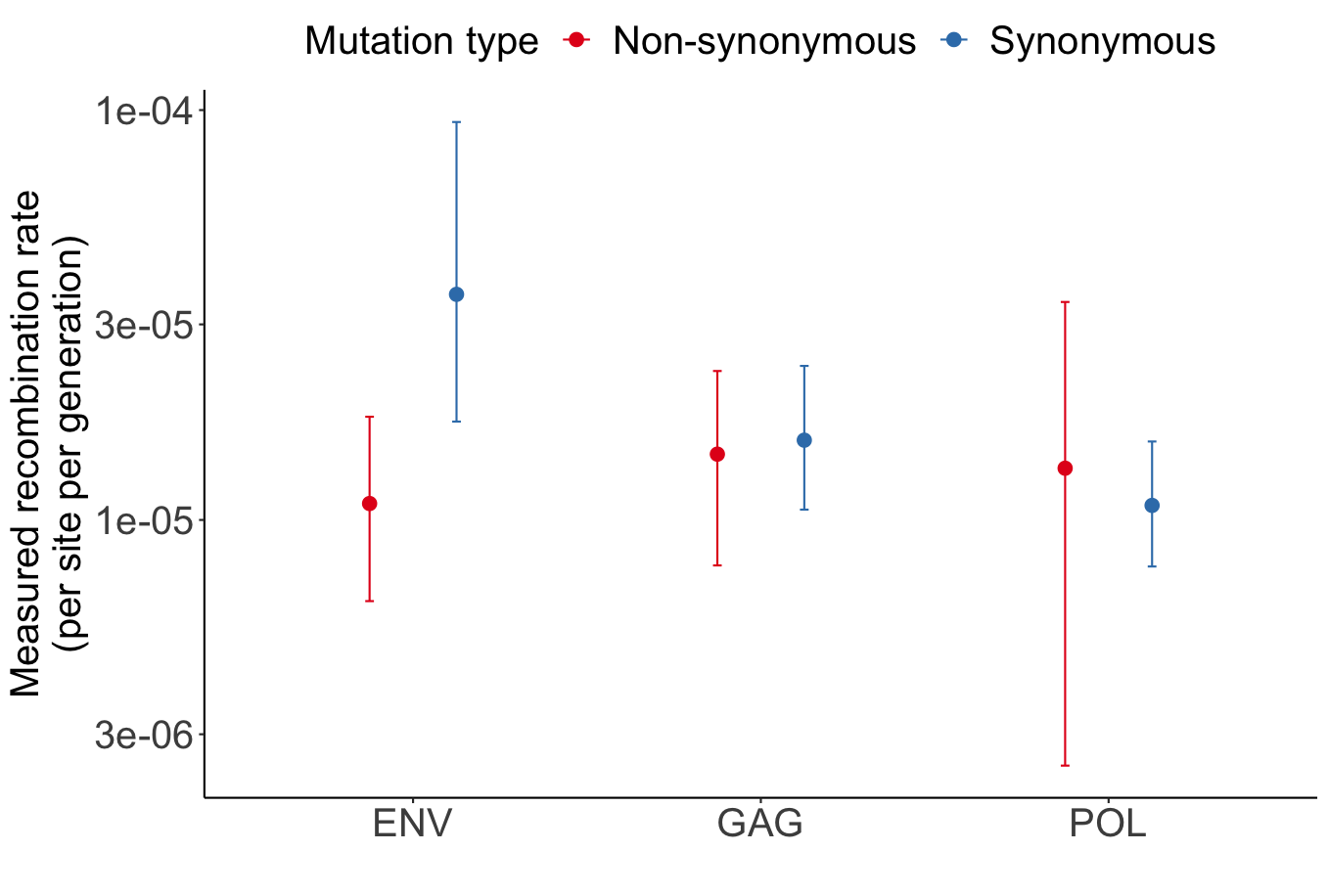


**Figure 4: Substantial differences in recombination rate in ENV by mutation type.** The recombination rate is estimated separately by mutation type (non-synonymous vs synonymous) of ENV, GAG and POL for all data points within the releant genes. Pairs of sites included in the ‘non synonymous’ group are restricted to pairs where the selection of the minor allele at both sites would result in a protein-level change, assuming no nucleotide substitutions at other sites within the same codon. Error bars represent 95% confidence intervals generated by bootstrap replicates. In *env*, we propose that a lower rate of measured recombination in pairs of sites at which there is a non-synonymous change signifies selection against recombinants.
