# Supplementary material for: Estimates of HIV-1 within-host recombination rates across the whole genome
Source: Virus Evol. 2025 Jul 25;11(1):veaf052. doi: 10.1093/ve/veaf052 (PMC12309388; doi:10.1093/ve/veaf052)
Supplement: suppTable1_veaf052 [file supptable1_veaf052.docx]

| HXB2 start | HXB2 end | Region type | Median rate (95% CI) **(** $\boldsymbol{\times10}^{\boldsymbol{-5}}\boldsymbol{)}$ | Genome-wide median rate (95% CI) **(** $\boldsymbol{\times10}^{\boldsymbol{-5}}\boldsymbol{)}$ | Lower/higher than genome average |
| --- | --- | --- | --- | --- | --- |
| 790 | 990 | Cold spot | -1.1 (-28--0.38) | 2.1 (1.6-2.7) | TRUE |
| 1290 | 1690 | Cold spot | 0.54 (0.27-0.81) | 1.8 (1.3-2.4) | TRUE |
| 3890 | 3990 | Cold spot | 11 (3.6-22) | 3.3 (2.3-4.9) | FALSE |
| 5140 | 5340 | Cold spot | 1 (0.48-1.7) | 2.1 (1.6-2.7) | TRUE |
| 7790 | 8190 | Cold spot | 0.48 (-106-3) | 1.8 (1.3-2.4) | TRUE |
| 1090 | 1189 | Hot spot | 12 (3.5-20) | 3.3 (2.2-5.6) | TRUE |
| 3190 | 3289 | Hot spot | 8.7 (3.9-16) | 3.3 (2.2-5.6) | TRUE |
| 4090 | 4289 | Hot spot | 6 (2.7-10) | 1.9 (1.4-2.5) | TRUE |
| 5890 | 6389 | Hot spot | 6.9 (4.1-9.6) | 2 (1.5-2.4) | TRUE |
| 8290 | 9090 | Hot spot | 3.5 (2.3-5) | 1.9 (1.5-2.3) | TRUE |
